# Supporting the evaluation of public and patient engagement in health system organizations: Results from an implementation research study

**DOI:** 10.1111/hex.12949

**Published:** 2019-08-02

**Authors:** Julia Abelson, Laura Tripp, Sujane Kandasamy, Kristen Burrows, on behalf of the PPEET Implementation Study Team

**Affiliations:** ^1^ Department of Health Research Methods, Evidence, and Impact, Faculty of Health Sciences McMaster University Hamilton Ontario Canada; ^2^ Centre for Health Economics and Policy Analysis (CHEPA) McMaster University Hamilton Ontario Canada; ^3^ Physician Assistant Education Program, Faculty of Health Sciences McMaster University Hamilton Ontario Canada

**Keywords:** evaluation instruments, evaluation studies, patient and public engagement, patient participation, research, surveys and questionnaires

## Abstract

**Background:**

As citizens, patients and family members are participating in numerous and expanding roles in health system organizations, attention has turned to evaluating these efforts. The context‐specific nature of engagement requires evaluation tools to be carefully designed for optimal use. We sought to address this need by assessing the appropriateness and feasibility of a generic tool across a range of health system organizations, engagement activities and patient groups.

**Methods:**

We used a mixed‐methods implementation research design to study the implementation of an engagement evaluation tool in seven health system organizations in Ontario, Canada focusing on two key implementation outcome variables: appropriateness and feasibility. Data were collected through respondent feedback questions (binary and open‐ended) at the end of the tool's three questionnaires as well as interviews and debriefing discussions with engagement professionals and patient partners from collaborating organizations.

**Results:**

The three questionnaires comprising the evaluation tool were collectively administered 29 times to 405 respondents yielding a 52% response rate (90% and 53% of respondents respectively assessed the survey's appropriateness and feasibility [quantitatively or qualitatively]). The questionnaires' basic properties were rated highly by all respondents. Concrete suggestions were provided for improving the appropriateness and feasibility of the questionnaires (or components within) for different engagement activity and organization types, and for enhancing the timing of implementation.

**Discussion and Conclusions:**

Our study findings offer guidance for health system organizations and evaluators to support the optimal use of engagement evaluation tools across a variety of health system settings, engagement activities and respondent groups.

## 1 INTRODUCTION

As citizens, patients and family members take on numerous and expanding advisory and leadership roles^1^, ^2^ in health system organizations, attention has turned to evaluating these efforts. Though the underlying goals for evaluation may differ,^3^ there is broad consensus about the need for stronger evidence to understand which engagement approaches work best, in which contexts and with what effects.^3^, ^4^ The number of tools and frameworks available to support the evaluation of public and patient engagement has increased steadily over the last number of years. Two recent systematic reviews identified 27 tools and over 100 metrics for evaluating patient engagement in research and health system decision making; however, most were developed without direct involvement of citizens or patients, or robust testing in different organizational contexts.^5^, ^6^

The Public and Patient Engagement Evaluation Tool (PPEET) was an early contribution to the engagement evaluation field. Developed by researchers and public and patient engagement practitioners, the goal of the PPEET was to provide a generic evaluation tool that could be used in a variety of health system organizations to evaluate and continuously improve the quality of public and patient engagement (PPE) activities. During its early development, the PPEET underwent usability testing with a limited group of respondents and end‐users (including patients and members of the public) who provided feedback on the structure, layout, comprehensibility, ease of use and overall utility of the survey.^7^ Given the widespread use of the tool since its launch in 2015, we set out to systematically assess its suitability for implementation across a broader range of organizational settings, engagement activities and user populations. The context‐specific nature of engagement practice requires evaluation tools to be carefully designed or chosen for optimal use. Results from this implementation research study have informed modifications to the PPEET and can offer guidance for health system organizations and evaluators to support the optimal use of engagement evaluation tools in a variety of health system settings.

### 1.1 Description of the Public and Patient Engagement Evaluation Tool (PPEET)

The PPEET is comprised of three surveys, each aimed at different target groups for evaluation. The *participant survey* aims to assess the processes, outputs and perceived impacts of engagement activities from the perspectives of citizens, patients and family members who may have various roles in the engagement process (e.g. consultant, advisor, partner). The *project survey* aims to evaluate the planning and execution of the engagement activity from the perspective of engagement implementers (e.g. engagement project leads, staff members). Finally, the *organization survey* aims to assess the culture and practices supporting public and patient engagement from the perspective of senior management and leadership in organizations (e.g. executive team members, board members). Each survey is structured around four core principles of “quality engagement” informed by an evidence synthesis and expert input: (a) integrity of design and purpose; (b) influence and impact; (c) participatory culture; and (d) collaboration and common purpose.^7^ The surveys include Likert‐scale and dichotomous questions, as well as open‐ended questions embedded throughout the survey.

## 2 RESEARCH DESIGN AND METHODS

We used a mixed‐methods implementation research design^8^, ^9^, ^10^ to study the PPEET's implementation in seven health system organizations in Ontario. Guided by Proctor et al's (2011) taxonomy of eight implementation outcomes (acceptability, adoption, appropriateness, feasibility, fidelity, cost, penetration and sustainability), we selected the two outcomes that most closely aligned with the stage of the tool's implementation (i.e. early) and our focus on further testing its usability, suitability and feasibility in different organizational contexts: (a) *appropriateness* (perception among stakeholders that the evaluation tool was relevant, useful and suitable for the target audience and organization); and (b) *feasibility* (the extent to which the tool could be implemented in a particular setting).^8^, ^11^ Each implementation outcome variable was assessed through a combination of quantitative and qualitative data collected from multiple sources using purposeful sampling.^12^ Quantitative (survey) data provided insights into the implementation of the PPEET and its appropriateness and feasibility from the perspective of the end‐user (see Appendix S1 for feedback survey). Qualitative data (open‐ended feedback provided through surveys, interviews and group discussions) supported efforts to understand, interpret and explain the quantitative data and to provide detail about the tool implementation process (appropriateness and feasibility).^13^ The study was reviewed and approved by the Hamilton Integrated Research Ethics Board (HIREB).

### 2.1 Recruitment of collaborating organizations

Study collaborators included early adopters of the PPEET (i.e. organizations using or interested in the tool immediately following launch), selected using the following inclusion criteria: organizations with different health system mandates (e.g. health‐care delivery vs provincial/local planning and quality improvement organizations), and organizations serving different types of communities or populations (e.g. large urban vs small northern community; disease‐specific vs population‐specific). The final group of seven collaborating organizations included large, urban, academic health science centres/networks (N = 3), provincial and/or regional health quality and integration organizations (N = 3), and one community‐based health services organization (N = 1; Table 1). Collaborating organizations committed to implementing each of the PPEET surveys (project and organization surveys once each, participant survey two to three times each). Each organization identified at least one staff member and at least one citizen/patient involved with PPE activities within the organization to join a Study Advisory Committee (SAC) to involve patients and organization staff at key stages of the research process. The committee included 16 members (5 patient partners and 11 staff members across the seven participating organizations) who were also invited to participate in different aspects of the study as survey respondents, key informant interviewees and/or debriefing call participants.

**Table 1:** Organizations participating in the implementation and evaluation of the Public and Patient Engagement Evaluation Tool

	Organization type	Mandate	Community served
HSO‐1	Health system organization	Provincial planning and quality improvement	Population‐specific (provincial)
HSO‐2	Health system organization	Provincial planning and quality improvement	Population‐ and disease‐specific (provincial)
HSO‐3	Health system organization	Regional planning and quality improvement	Population‐specific (regional)
AHSC‐1	Academic health science centre/network	Health system delivery	Population‐specific (regional, paediatric)
AHSC‐2	Academic health science centre/network	Health system delivery	Population‐specific (regional)
AHSC‐3	Academic health science centre/network	Health system delivery	Population‐specific (regional)
CHSO‐1	Community‐based health services organization	Health service delivery	Population‐specific (regional)

### 2.2 Recruitment of survey respondents

In consultation with the research team, staff members within each of the collaborating organizations, identified the engagement activities that would be evaluated and oversaw the recruitment of citizens, patients, family members and staff to complete the surveys. The research team had no direct involvement in the recruitment process or contact with survey participants. All individuals involved with each of the selected engagement activities were recruited to participate in the survey. Recruitment methods varied by organization; each organization documented their recruitment process including the number of individuals approached to complete a survey and the recruitment strategies used.

### 2.3 Data collection

Each of the three PPEET questionnaires contains a number of Likert‐scale questions (Participant: 14 questions; Project: 24 questions; Organization: 27 questions) and a small number of open‐ended questions (Participant: 4 questions; Project: 4 questions; Organization: 5 questions). A separate feedback survey was added to each evaluation questionnaire to assess respondents' perspectives on the appropriateness (Questions 1‐5 and 7) and feasibility of the questionnaire (Questions 6 and 7) using both scaled and open‐ended questions (see Appendix S1).

Organizations could opt to collect the survey data themselves or have the data collected directly by the research team through an online survey platform (LimeSurvey). Organizations collecting data shared it securely with the research team. When the online survey platform was used, the research team programmed the survey and shared a survey link with the organization, which was distributed to potential respondents. Completed surveys were stored directly on the secure online platform. No identifying information was collected through the survey, ensuring the anonymity of respondents.

### 2.4 Post‐implementation activities

Following each implementation, a report summarizing the results of the survey was shared with the partner organization. Organizations were encouraged to share evaluation results with the engagement participants. A member of the research team completed a 15‐ to 30‐minute debriefing call with the PPE project lead and/or the public/patient representative in each partner organization to discuss the PPE activity evaluated and the process used to implement the survey (eg recruitment strategies, dissemination of survey results). During these calls, feedback on the appropriateness and feasibility of the tool was collected from the implementer's perspective (e.g. survey implementation time, concerns with the survey, feedback from users). Additional feedback on the tool implementation process was collected through three advisory committee meetings held over the 1.5‐year project.

### 2.5 Data analysis

PPEET feedback survey data were analysed using descriptive statistics for the quantitative components and qualitative thematic analysis for the open‐ended questions.^14^ Analysis was completed initially at the aggregate level and subsequently by type of organization and by type of engagement activity (where applicable). Feedback results were compared by survey, activity and organization type using two‐tailed Fisher's exact tests (*P‐*values of <.05 were considered statistically significant). Individual participant responses to each open‐ended question were collated by question and coded to common themes across questions. Coding reliability was ensured through an iterative process where the primary coder (SK) and principal author (JA) met several times during the development and application of the coding scheme to review independent coding of selected data excerpts and resolve inconsistencies. Coding reliability was further corroborated by a third team member (LT) in the later stages of analysis. The results were shared with the Study Advisory Committee during an in‐person meeting for the purposes of member‐checking and additional discussion and reflection.

## 3 RESULTS

Each of the PPEET instruments was implemented in at least four of the seven partner organizations and between 1 and 7 instances within each (Table 2). Results for each survey type are presented separately, below. Where appropriate, results are presented by engagement activity and organization type. Engagement activity types included the following: (a) short‐term consultations with patients only or patients and organization staff; (b) on‐going engagement activities with patient partners in an advisory role; and (c) knowledge exchange activities such as patient summits and fairs (Table 3). Qualitative themes are presented, by survey type, in Table S1.

**Table 2:** Implementation of the Public and Patient Engagement Evaluation Tool and feedback questions during the implementation study

Survey	Organizations implementing the survey	Number of times implemented (range across orgs)	Surveys distributed	Completed surveys (response rate)	Completed a feedback survey (response rate)*	Provided written comments in feedback survey (response rate)
Participant	7	17 (1‐7)	271	152 (56%)	131 (86%)	70 (53%)
Project	4	7 (1‐4)	18	16 (89%)	16 (100%)	15 (94%)
Organization	5	5**	116	43 (37%)	43 (100%)	15 (35%)
TOTAL	n/a	29	405	211 (52%)	190 (90%)	100 (53%)

* Respondents skipped no more than 2 of the feedback questions.

** No range provided; survey implemented once in each organization.

**Table 3:** Number engagement activities evaluated with participant and project surveys, by organization type

Activity type	Organization type					
	Academic health science centres/networks		Provincial and regional health system organizations		Community‐based health system organization	
	Participant	Project	Participant	Project	Participant	Project
Long‐term activities	3	3	3	0	0	0
Short‐term activities	7	2	2	2	0	0
KTE activities	0	0	1	0	1	0
TOTAL	10	5	6	2	1	0

### 3.1 Participant survey

Each partner organization implemented the participant survey at least once and as many as seven times over the study period. In total, 271 participant surveys were administered across the 7 organizations and 17 engagement activities and 152 surveys were completed. Of these, 131 respondents completed at least two of the feedback questions, which are the focus of our analysis (Table 2). Demographic characteristics of feedback survey respondents are presented in Table 4. Approximately one‐third of respondents (34.4%) chose not to reply to at least one demographic question, leading to an incomplete profile of study participants. Of those who provided demographic information, just under 25% were 58 or older and almost two‐thirds were female (63.4%). Respondents were well‐educated (56.5% had completed at least a university degree); just under half reported working either full‐ or part‐time (45.8%).

**Table 4:** Respondent demographics, participant survey

Characteristics	N (n = 131)	%
**Year of birth**		
Prior to 1960 (58 y of age or older)	32	24.4
1960‐1980 (37‐57 y of age)	54	41.2
1981‐2000 (17‐36 y of age)	11	8.4
2000 or later (16 or younger)	0	0
No response	34	26.0
**Gender**		
Male	34	26.0
Female	83	63.4
Other	3	2.3
No response	11	8.4
**Highest level of education completed**		
High school	9	6.9
Community college	19	14.5
Technical school	7	5.3
Bachelor's degree (Arts, Science, etc)	38	29.0
Postgraduate training or professional or graduate degree	36	27.5
Prefer not to answer/no response	22	16.8
**Current employment status**		
Working for pay full‐time (including on strike and any form of leave)	46	35.1
Working for pay part‐time (including retired part‐time, homemaker part‐time)	14	10.7
Student (includes students working part‐time)	2	1.5
Retired	28	21.4
Not in labour force, able to work	3	2.3
Not in labour force, unable to work	14	10.7
Homemaker	3	2.3
Prefer not to answer, no response	21	16.0
**Annual income before taxes and deductions**		
Less than $20 000	12	9.2
Between $20 000 and $39 999	7	5.3
Between $40 000 and $59 999	24	18.3
Between $60 000 and $79 999	14	10.7
More than $80 000	29	22.1
Prefer not to answer	45	34.4

#### 3.1.1 Quantitative results

Overall, the quantitative responses yielded favourable views towards the survey (Table 5). Nearly all respondents indicated that the survey (96.9%) and its layout (97.7%) were easy to use and that the instructions were clear and helpful (94.7%). Most found the questions easy to understand (87.0%). Just over two‐thirds of respondents did not think any important questions were missing (68.7%).

**Table 5:** Feedback results across all PPEET surveys

Feedback question	Response	Survey			*P*‐value
		Participant (n = 131)	Project (n = 16)	Organization (n = 43)	
Overall, survey was easy to use	Yes	127 (96.9%)	14 (87.5%)	41 (95.3%)	.09
	No	3 (2.3%)	2 (12.5%)	2 (4.7%)	
	No response	1 (0.8%)	0 (0%)	0 (0%)	
The instructions were clear and helpful	Yes	124 (94.7%)	14 (87.5%)	41 (95.3%)	.14
	No	4 (3.1%)	2 (12.5%)	2 (4.7%)	
	No response	3 (2.3%)	0 (0%)	0 (0%)	
The layout was easy to use	Yes	128 (97.7%)	14 (87.5%)	41 (95.3%)	**.04** *
	No	1 (0.8%)	2 (12.5%)	0 (0%)	
	No response	2 (1.5%)	0 (0%)	2 (4.7%)	
The questions were easy to understand	Yes	114 (87.0%)	5 (31.3%)	39 (90.7%)	**<.001** *
	No	14 (10.7%)	11 (68.8%)	3 (7.0%)	
	No response	3 (2.3%)	0 (0%)	1 (2.3%)	
There were important questions missing	Yes	28 (21.4%)	1 (6.3%)	6 (14.0%)	.27
	No	90 (68.7%)	13 (81.3%)	32 (74.4%)	
	No response	13 (9.9%)	2 (12.5%)	5 (11.6%)	
Results will be useful for the organization	Yes	—	12 (75.0%)	39 (90.7%)	.27
	No	—	2 (12.5%)	2 (4.7%)	
	No response	—	2 (12.5%)	2 (4.7%)	

* Statistically significant at *P* = .05.

The survey was also viewed positively across all types of engagement activities with some minor differences. Respondents who participated in *short‐term activities* gave slightly higher ratings than those in the other two groups. Respondents who participated in *knowledge translation and exchange (KTE) activities* stated most frequently that important questions were missing (25.0%) compared to the other two groups (Table 6), although this difference was not statistically significant. Results were quite uniform across the three organization types. Individuals from provincial and regional health system organizations were significantly more likely to report that important questions were missing (32.2%; *P* = .02) and those from community‐based health system organizations stated most frequently, although not statistically significant, that the questions were hard to understand (25.0%) (Table 7).

**Table 6:** Participant survey feedback results, by engagement activity type

Feedback question	Response	Activity type			*P*‐value
		Long‐term activities (n = 50)	Short‐term activities (n = 37)	KTE activities (n = 44)	
Overall, survey was easy to use	Yes	48 (96.0%)	37 (100%)	42 (95.5%)	.78
	No	2 (4.0%)	0 (0%)	1 (2.3%)	
	No response	0 (0%)	0 (0%)	1 (2.3%)	
The instructions were clear and helpful	Yes	46 (92.0%)	36 (97.3%)	42 (95.5%)	.55
	No	2 (4.0%)	0 (0%)	2 (4.5%)	
	No response	2 (4.0%)	1 (2.7%)	0 (0%)	
The layout was easy to use	Yes	49 (98.0%)	37 (100%)	42 (95.5%)	.62
	No	0 (0%)	0 (0%)	1 (2.3%)	
	No response	1 (2.0%)	0 (0%)	1 (2.3%)	
The questions were easy to understand	Yes	44 (88.0%)	33 (89.2%)	37 (84.1%)	.40
	No	5 (10.0%)	2 (5.4%)	7 (15.9%)	
	No response	1 (2.0%)	2 (5.4%)	0 (0%)	
There were important questions missing	Yes	11 (22.0%)	6 (16.2%)	11 (25.0%)	.68
	No	33 (66.0%)	27 (73.0%)	30 (68.2%)	
	No response	6 (12.0%)	4 (10.8%)	3 (6.8%)	

**Table 7:** Participant survey feedback results, by organization type

Feedback question	Response	Organization type			*P*‐value
		Academic health science centres/networks (n = 60)	Provincial and regional health system organizations (n = 59)	Community‐based health system organization (n = 12)	
Overall, survey was easy to use	Yes	60 (100%)	56 (94.9%)	11 (91.4%)	.08
	No	0 (0%)	2 (3.4%)	1 (8.3%)	
	No response	0 (0%)	1 (1.7%)	0 (0%)	
The instructions were clear and helpful	Yes	57 (95.0%)	56 (94.9%)	11 (91.4%)	.11
	No	0 (0%)	3 (5.1%)	1 (8.3%)	
	No response	3 (5.0%)	0 (0%)	0 (0%)	
The layout was easy to use	Yes	59 (98.3%)	57 (96.6%)	12 (100%)	.54
	No	0 (0%)	1 (1.7%)	0 (0%)	
	No response	1 (1.7%)	1 (1.7%)	0 (0%)	
The questions were easy to understand	Yes	54 (90.0%)	51 (86.4%)	9 (75%)	.07
	No	3 (5.0%)	8 (13.6%)	3 (25%)	
	No response	3 (5.0%)	0 (0%)	0 (0%)	
There were important questions missing	Yes	7 (11.7%)	19 (32.2%)	1 (8.3%)	**.02** *
	No	45 (75.0%)	36 (61.0%)	11 (91.4%)	
	No response	8 (13.3%)	4 (6.8%)	0 (0%)	

* Statistically significant at *P* = .05.

#### 3.1.2 Qualitative results

Over half of respondents (Table 2) provided written comments about the questionnaire as a complement to the scaled questions. Much of the written feedback supported the quantitative results and reinforced views that the questions were appropriate, clear and relevant and that the survey was thorough and straightforward (see Appendix S1). The participant survey appeared to work equally well in both health‐care delivery and provincial/regional health organization settings. The only activity evaluated within a community‐based health‐care delivery organization was a KTE activity; therefore, additional work is needed to confirm the appropriateness and feasibility of the survey in this specific context.

Major areas of feedback focused on the appropriateness and feasibility of the survey and specific sections or items within it, and the need for the tailoring to specific contexts. Minor areas of feedback included requests for additional questions to allow for more detailed exploration of the engagement process, minor wording changes and a request for “not applicable” response options.

##### Appropriateness of the questionnaire for different types of engagement activities

Although those who participated in short‐term engagement activities felt that questions in this survey “were valid and in‐scope of the activity” (AHSC‐3, Activity 13), many others questioned its appropriateness for longer‐term and KTE activities.

For respondents who were involved in longer‐term engagement activities such as councils and committees, the survey presented challenges in determining the unit of engagement to be evaluated (i.e. a specific activity or meeting or the engagement process as a whole) and how to assess the features of multiple meetings when the quality of engagement might vary from one meeting to the next. This led a number of respondents to state that the survey was better suited to a one‐time activity, a view that was shared by the Study Advisory Committee.

This survey is evaluating approximately 10 meetings and doesn't lend itself well to that assessment…some meetings diversity of opinions was sought, sometimes not. (AHSC‐1, Activity 3)

The questions seem better directed to a one‐time, one‐topic focus group rather than an ongoing council. (HSO‐3, Activity 12)

For KTE activities, respondents cited the challenge of questions that were “very broad, hard to relate to the [activity] format/context” (HSO‐1, Activity 2) and, correspondingly, the need for them to be “more detailed and specific” (CHSO‐1, Activity 11).

##### Relevance of specific sections or questions and the need for tailoring

A small number of questions generated insightful discussions among respondents and within the Study Advisory Committee. A notable example was the statement “As a result of my participation in this activity, I have greater trust in [organization]”. Some respondents felt this question was difficult to answer and that it would “depend on whether we get responses” to the feedback provided (AHSC‐1, Activity 4). Others did not feel the question “resonated with the work of an advisor” (AHSC‐1, Activity 3) or they did not “understand the relevance of the trust question” further explaining that “I didn't distrust [the organization] in the first place and attending [committee meetings] would not impact my organizational trust even if I had issues” (AHSC‐1, Activity 3). To these respondents, trust was formed (and broken) at the individual clinic/practitioner level and not within patient engagement activities. Tool implementers agreed that trust was a complex construct to measure and likely influenced by why individuals became engaged in the first place. Many implementers felt the trust question should be removed from the survey.

The optional demographics section at the end of the questionnaire also generated considerable feedback and discussion. This section includes standard questions that collect information about respondents' age, gender, income, education, membership in a vulnerable group (e.g. recent immigrants), and employment history in the health‐care sector. Many participants understood the purpose of including these questions and recommended minor modifications (e.g. additional education level categories; clarifying personal vs household income and adding additional marginalized groups). Some, however, felt that these questions were intrusive, overly personal and irrelevant to their engagement activities. Some of this discomfort stemmed from uncertainty about “what [organizers] would do with this information” (HSO‐1, Activity 2) especially when it came to questions about income and age. This also likely explains why over a third of respondents did not share their income information in this section of the survey. There were concerns that responses to these questions might be used to prioritize the perspectives of some over others. A particular concern raised by a respondent involved in a long‐term activity was how the survey findings would be anonymized as “the questions asked compared to the demographics of [those participating] make it very easy [for the respondent] to be identified” (HSO‐2, Activity 6). Another noted that these types of demographics did not seem appropriate for the particular setting they were involved in, and that questions related to individual's health status and experience with the health‐care system would be more relevant.

From the tool implementers' perspective, collaborating partners acknowledged the importance of collecting demographic information to provide insights about the diversity of those involved in their engagement activities. However, some echoed the concerns shared by participants regarding how the information would be used and their ability to protect respondents' anonymity, particularly in the context of committees where small numbers of patient partners are typically involved. Others raised concerns about respondent burden given that patients are often asked these types of questions in health‐care settings. To address these concerns, recommendations were made for being more selective and purposeful in the use of the demographic questions with rationales provided for the inclusion of these questions.

Preface this as to why you're [collecting] this, [that] you want to be sure you're hitting all demographics [with your participant group]. That could maybe help to ease people's fears.

##### Requests for additional questions and response options

A number of respondents used the open‐ended comments section to request additional questions or themes that could be explored about the engagement experience. Notably, many requested the inclusion of additional comment sections, some requesting open‐ended comment boxes to accompany each item in the scale. The inclusion of comment boxes was viewed as an important vehicle for providing context and nuance to their scaled responses.

Without opportunity for comment, my views would not have been explained. (HSO‐1, Activity 2)

The thing I appreciate most is having text‐boxes…there's no survey on earth that's going to be able to ask all the right questions and even if they do…you can't share the nuances of your experience that way. (Patient partner member of Study Advisory Committee)

### 3.2 Project survey

The project survey was implemented by four separate organizations (1‐4 implementations each). Both short‐ and long‐term engagement activities were evaluated using the tool. The response rates for this survey were much higher than for the participant survey (89%); all respondents completed feedback questions and 94% contributed open‐ended comments). Organizations had some difficulty identifying engagement activities and initiatives to evaluate using the project survey resulting in a fewer implementations across and within organizations. As a result, data were not analysed at the level of engagement or organization type.

#### 3.2.1 Quantitative results

Generally, respondents felt the survey was easy to use (87.5%) and the instructions were clear and helpful (87.5%), and few felt important questions were missing (6.3%). Across all surveys, the project survey respondents were least likely to feel the layout was easy to work with (87.5%, *P* = .04). Although three‐quarters of respondents (75%) reported that the results of this survey would be useful for their organization, over two‐thirds indicated that questions were not easy to understand (68.8%) which was significantly higher than the PPEET participant and organization questionnaires (*P* < .001), suggesting the need for significant revisions (Table 5).

#### 3.2.2 Qualitative results

Almost all respondents (Table 2) provided written comments about this questionnaire as a complement to the scaled questions (see Appendix S1). Major feedback focused on the relevance of the survey for certain engagement activities (i.e. larger‐ vs smaller‐scale activities), identifying the most appropriate people in the organization to complete the survey and questions about the most appropriate time for survey administration to balance the desire to evaluate within a reasonable time period against the need to allow sufficient time to pass for longer‐term assessments of how the input would be used or make a difference .

It is a bit challenging for project staff to answer on behalf of leadership in the organization. (HSO‐2, Activity 10)

Timing of doing the questionnaire is key (couldn't answer many questions because we hadn't moved along in the process) (CHSO‐1, Activity 23)

[It's] too soon to say if [participant] input will be used. (AHSC‐3, Activity 15)

Other feedback focused on the lack of clarity of specific questions, the repetitive nature of the survey and the applicability of certain questions to their engagement context (e.g. if they did not collaborate with other organizations, questions about collaboration were not relevant).

I didn't find the questions entirely easy to understand ‐ I felt I had to read each carefully to ensure I knew what they were asking. (AHSC‐3, Activity 13)

Some of the questions were open to interpretation and too vague to answer specifically. (AHSC‐3, Activity 22)

Some questions seemed very similar and it wasn't clear what the distinction was between them. (AHSC‐3, Activity 15)

The length of the survey was of concern to some respondents who felt it was “onerous to complete” (HSO‐2, Activity 9). Respondents felt that the number of questions overall could be reduced as well as the number of open‐ended questions more specifically. A desire for questions to “focus a bit more on more of a micro level” (HSO‐2, Activity 10) was also communicated to make them easier for staff to respond to. A member of the Study Advisory Committee noted that the length of the survey presented challenges for staff to find the time to complete it and recommended using the survey as a guide for conversations with staff about the engagement activity. The survey's utility in encouraging best practices was also noted:

it's kind of human nature…if we put [an item] on the checklist and we know that we're being evaluated by it, we're sort of directly and indirectly pushing people to do best practice if you know you're going to be evaluated on it at the end of the day, right?

### 3.3 Organization survey

The organization survey was implemented by 5 of the 7 organizations but achieved the lowest response rate of the three (37%); all respondents completed feedback questions, and 35% provided open‐ended comments). Four of the organizations used it to evaluate their public and patient engagement work across the organization. One organization used it to evaluate engagement within a specific part of the organization.

#### 3.3.1 Quantitative results

As with the other two surveys, most respondents felt this survey was easy to use (95.3%), the instructions were clear and helpful (95.3%), and the layout was easy to use (95.3%). Some felt important questions were missing (14.0%) and most found the questions easy to understand (90.7%). Almost all (90.7%) thought the results would be useful for their organization (Table 5).

Overall, respondents from health‐care delivery organizations were positive about the survey with few individuals indicating any concerns with the survey within the quantitative questions. Albeit not statistically significant, respondents from provincial and regional health system organizations indicated more often that there were important questions missing from the survey (27.3%) and that the questions were not easy to understand (18.2%) (Table 8).

**Table 8:** Organization survey feedback results, by organization type

Feedback question	Response	Organization type			*P*‐value
		Academic health science centres/networks (n = 26)	Provincial and regional health system organizations (n = 11)	Community‐based health system organization (n = 6)	
Overall, survey was easy to use	Yes	26 (100%)	10 (90.9%)	5 (83.3%)	.15
	No	0 (0%)	1 (9.1%)	1 (16.7%)	
	No response	0 (0%)	0 (0%)	0 (0%)	
The instructions were clear and helpful	Yes	25 (96.2%)	10 (90.9%)	6 (100%)	.64
	No	1 (3.8%)	1 (9.1%)	0 (0%)	
	No response	0 (0%)	0 (0%)	0 (0%)	
The layout was easy to use	Yes	24 (92.3%)	11 (100%)	6 (100%)	1.0
	No	0 (0%)	0 (0%)	0 (0%)	
	No response	2 (7.7%)	0 (0%)	0 (0%)	
The questions were easy to understand	Yes	24 (92.3%)	9 (81.8%)	6 (100%)	.21
	No	1 (3.8%)	2 (18.2%)	0 (0%)	
	No response	1 (3.8%)	0 (0%)	0 (0%)	
There were important questions missing	Yes	2 (7.7%)	3 (27.3%)	1 (16.7%)	.30
	No	21 (80.8%)	8 (72.7%)	3 (50%)	
	No response	3 (11.5%)	0 (0%)	2 (33.3%)	
Results will be useful for the organization	Yes	25 (96.2%)	9 (81.8%)	5 (83.3%)	.60
	No	1 (3.8%)	1 (9.1%)	0 (0%)	
	No response	0 (0%)	1 (9.1%)	1 (16.7%)	

#### 3.3.2 Qualitative results

Just over one‐third (35%) of those who completed the feedback questions on the organization questionnaire provided open‐ended comments, compared to over 50% and 90% for the other surveys (Table 2). Major feedback focused on the survey's suitability for certain types and sizes of organizations, identifying the most appropriate people in the organization to complete it as well as ensuring that the questions are relevant to the intended respondents (see Appendix S1). Minor feedback focused on suggestions for clarifying specific wording, including more open‐ended response options and not applicable response categories and a time estimate for completing the survey in the instructions.

On the theme of the appropriate organization type, the organization survey was critiqued for not adequately capturing the approach to engagement in an organization with a strong community orientation.

Engagement is part of the fabric of a true community organization. This survey puts the concept of public and patient engagement as a separate entity, it is not and therefore the questions are not in some instances relevant. (CHSO‐1)

The relevance of the survey to smaller‐sized organizations was also questioned—specifically organizations where there are fewer resources to dedicate to engagement, which could influence the responses to the survey questions. A related question about the framing of the survey was raised by one of the hospital sector respondents who noted that the survey seemed to view engagement as needing “additional resources or infrastructure rather than as a thread through existing structures” (AHSC‐3).

Identifying the “right” individuals to participate in the survey and ensuring that the questions were relevant to range and type of respondents who might complete it within organizations was also identified as important. For example, one individual felt that “some questions were also hard to answer because they're out of the scope of what a manager would know” (HSO‐1).

As with the project survey, a number of questions were cited for improved clarity, framing or contextualizing. Notably, greater precision was requested about the level within the organization at which the survey is being targeted. For example, it was not clear if they should be responding “specifically about what's done on a programmatic level, vs portfolio or [organization] at large” (HSO‐1). Others noted that the survey did not always fit with how engagement was operationalized in their context. For example, two hospital respondents spoke of how the question assumes that engagement reports are available and sent out, and this is not always the case (either because they are shared in other ways or because they are not available).

## 4 DISCUSSION

Our results provide insights into the appropriateness and feasibility of a set of three questionnaires for implementation in a variety of health system organizations to evaluate different types of engagement activities. Overall, the questionnaires comprising the PPEET were viewed by survey respondents and evaluation practitioners as useful, easy to administer and reasonably complete. While these findings are consistent with the results from the early usability testing results that informed the development of the first version of the PPEET,^7^ the more comprehensive testing of the tool undertaken through this implementation study has improved the tool in important ways since its release in 2015. In addition to generating critical feedback on several aspects of the questionnaires (e.g. questions missing or difficult to understand), valuable guidance was also provided for increasing the tool's appropriateness for specific engagement activities and respondent groups, and for enhancing feasibility of implementation.

The tool revisions and related guidance documents informed by this implementation research study are now available (see https://ppe.mcmaster.ca). Across‐the‐board modifications include both formatting and design revisions. Expanded guidance documents were developed for both respondents and tool implementers to tailor surveys to appropriate engagement activities and respondent groups. More comprehensive modifications have been made to the participant and project surveys in response to our study findings. The *participant questionnaire* was split into two discrete modules to address concerns about the appropriateness of a single instrument for both short‐ and longer‐term engagement activities. The *project questionnaire* was split into three modules, each to be used at different times during the engagement implementation process: (a) during the planning of the engagement activity; (b) immediately following the activity; and (c) 3‐6 months following the activity. These changes have shortened the surveys and ensure that the right questions are asked at the right time. Only minor changes were made to the *organization questionnaire* mostly focused on an expanded guidance document to respond to lack of clarity about its purpose and to emphasize its purpose in relation to the other two surveys.

### 4.1 Study contributions, strengths and limitations

The number of engagement evaluation tools and frameworks is growing. Associated with this trend are encouraging examples of more rigorous tool development and testing, with the direct involvement of patients.^6^, ^15^ However, studies focused on assessing the robustness of engagement evaluation tools in a real‐world setting or in the specific context of health‐care delivery and system planning organizations are still few in number.^16^ We believe this study is one of the first efforts to systematically assess the appropriateness and feasibility of an engagement evaluation tool across a range of organizational settings, engagement activities and user populations, using a recognized framework and selected outcome variables.^8^, ^11^ Our results offer several key messages to the engagement evaluation field as it continues to grow and mature. First, our findings demonstrate the importance of continuous and on‐going field testing of evaluation tools particularly in the engagement field where methods and approaches are continuously evolving, where organizations are at different stages of maturity in their public and patient engagement practice, and where organizational culture and context play such important roles in shaping approaches to engagement and its evaluation. One of the study's key findings, for example, that the participant survey worked well for short‐term engagement activities, but was a poor fit for longer‐term or on‐going engagement activities, illustrates this point well. As the field matures and organizations develop greater sophistication, evaluation methods and tools will need to adapt to these changing conditions.

Second, our findings highlight the importance of attending to engagement participants' strong desire to provide feedback in a variety of ways (i.e. through closed‐ and open‐ended questions), to ensure their feedback can be provided anonymously and that it will not be dismissed or given lower priority based on who they are. This relates to a third key message regarding the need for continued attention to the core principles of high‐quality engagement and that these should be extended to its evaluation (e.g. match engagement goals to methods and recruitment, ensure clarity of communication through all stages, including how the input will be used and the sharing of key reports on the engagement process).^17^, ^18^, ^19^ In adhering to principles of good engagement practice, organizations must not only be willing to evaluate their engagement activities but to share the feedback collected and plans for acting on it. The dominance of this theme in respondents' open‐ended comments highlights its importance to organizations with PPE mandates. If engagement participants feel that their contributions are not being given serious consideration or cannot be traced to something tangible, their interest and commitment will wane.

Our study is not without limitations. Despite our efforts, the organization and project tools were not implemented in each of our collaborating organizations, limiting our ability to robustly explore differences by organizations or engagement activity type. Further, the participant survey was only implemented once within a community‐based health services organization with one type of engagement activity, limiting our ability to generate conclusive results about tool implementation specific to this setting. A further limitation was the structure of the feedback survey. The feedback survey included one open‐ended question and 5‐6 dichotomous questions (yes/no) which only prompted for qualitative responses to explain negative responses. Feedback questions were guided by previous work in this area,^7^ and consultations with engagement practitioners (through the Study Advisory Committee). While not including additional open‐ended questions and not using scaled questions limited the amount of information available on the survey's appropriateness and feasibility, this decision was balanced against concerns that longer and more complex questions at the end of the main evaluation survey would be burdensome for respondents to complete. The seven health system organizations that chose to participate in this study were early and willing adopters of the PPEET (two of the organizations had used at least one of the questionnaires prior to joining the study). While it is likely that these “early adopter” characteristics played some role in framing their perspectives, it is not clear how. Similarly, the public and patient respondents to the participant survey may have also differed in some ways based on their experiences. Unfortunately, we did not collect information about respondents' length of experience within the organization or with other engagement activities to be able to explore these relationships in our study; questions to elicit this information have been added to the new participant questionnaire. Our participant demographic data indicate that our participant sample tended to be highly educated, with a large number of middle‐ to older‐age women. Here again, we are limited in the conclusions we can draw from this information given the large number of respondents who did not reply to the demographic questions, and given our currently limited understanding of the composition of patient partner communities in the study jurisdiction or in health systems more broadly. Due to the lack of comprehensive, systematically collected data about patient partner communities, we cannot state confidently whether our study respondents are representative or reflective of patient engagement participants and partners in Ontario or elsewhere. Finally, the arm's‐length and consultative relationship we established with our collaborating partners resulted in some weaknesses in study execution. Although we consulted with organizations in the selection of engagement activities for evaluation, final decisions were left to them which, in two instances, resulted in the selection of engagement activities that, in our view, were poorly matched to the evaluation tool and inappropriately implemented. While these observations were useful in confirming the tool's lack of fit for these types of activities, this experience could have been avoided. Similarly, the research team had no control over the mode of administering the survey, including how participants were recruited, and the timing of the administration of the surveys within each organization, which likely affected survey response rates, and potentially, the feedback obtained on the tool itself. While these are limitations to the current study, they offer useful guidance for the conduct of future evaluation studies in the field.

## 5 CONCLUSION

This study provides insight into how the PPEET (and other evaluation tools) can be more optimally implemented to evaluate a range of public and patient engagement activities within a variety of health system organizations. Working closely with different health organizations to understand the appropriateness and feasibility of the PPEET surveys provided valuable information about how to improve the PPEET in simple yet effective ways. We look forward to continued efforts to develop and rigorously assess the PPEET and other engagement evaluation tools to support and continuously improve the quality of engagement work carried out in health system organizations.

## CONFLICT OF INTEREST

The authors have no conflicts of interest to report.

## Data Availability

The data that support the findings of this study are available from the corresponding author upon reasonable request.
