# Supplementary material for: Supporting the evaluation of public and patient engagement in health system organizations: Results from an implementation research study
Source: Health Expect. 2019 Aug 2;22(5):1132–43. doi: 10.1111/hex.12949 (PMC6803403; doi:10.1111/hex.12949)
Supplement: Supplementary file 1 [file HEX-22-1132-s001.docx]

**Supplementary Table: Feedback Survey Results (Qualitative themes), by survey type**

| **QUESTION** | **SURVEY** | **THEMES** |
| --- | --- | --- |
| Questions were easy to understand | Organization | N = 4   - Question design: inappropriate response options - Relevance to organization - Purpose of tool: evaluating a program, department or organization - Sample: are the right individuals are completing the survey |
|  | Project | N = 10   - Applicability of questions: less relevant for smaller engagement activities, doesn’t fit the specific activity - Question design: confusing, vague, similar - Clarification of terminology: engagement process’, too technical - Survey implementation: survey timing |
|  | Participant | N = 58   - Structure of tool: formatting, length - Function of tool: ease of use - Question design: broad, vague - Clarification of terminology: participation |
| Using this questionnaire will be useful for our organization | Organization | N = 3   - Need for additional questions about impact and outcomes - Uncertain |
|  | Project | N = 3   - More useful for large scale engagements - If implemented at the correct time within the process - Uncertain |
|  | Participant | *Not applicable* |
| There are important questions missing | Organization | N = 9   - Questions to add: - Frequency of contact with patient advisors - Specifics of engagement activities (e.g., recruitment strategies; barriers and facilitators; methods used; governance of programs) - Personal perspectives on engagement activities - Don’t solely focus on “patient” engagement but also community engagement - More open-ended questions |
|  | Project | N = 1   - Questions to add: Intended and actual impacts of engagement |
|  | Participant | N = 64   - General feedback: Questions are too generic, leading (too many started with the response option “yes” instead of “no”) - Questions to add: Add additional response options to questions asking about minority groups (e.g., add LGBTQ); add a question to capture if participants feel as though they are working as part of a team vs. a mandated requirement - Correct application of tool: usefulness for ongoing activities vs. single, one-time activities; is the tool useful for councils/committees that are just forming |
| Identify one way the survey can be improved | Organization | N = 8   - Survey questions: add ‘not applicable’ response option; be clear what organization level the survey is assessing - Survey format: add length information to beginning - Sampling: carefully consider who should receive the survey |
|  | Project | N = 8   - Survey questions: add ‘not applicable’ response option; avoid generalization in questions – add name of the group or organization; ensure language is appropriate for target audience - Survey format (add a status bar to know how far into the survey you are; ensure response options are always visible on the page) - Reduce the length of the survey - Implementation: carefully consider implementation timing |
|  | Participant | N = 42   - General feedback on questions: satisfied with questions; length was appropriate; questions were too vague, not specific - Suggested questions: What are perceived obstacles to change; question about ongoing work - Demographic questions: Remove demographic questions, especially income and education – concern about segregating patients/caregivers by social class; Don’t isolate “healthcare” employment with unless ask all fields of employment; Expand education questions/ add additional options - Response options: If “do not agree” is an option, add comment box to allow explanation; Give examples - Survey function: Add way of knowing how far along/# questions in survey - General feedback: Reduce survey length; Timing of survey – i.e. provide before leaving activity; Be clear how survey results will be used and in what timeframe |
| Additional Comments: | Organization | N = 6   - Survey questions: need to improve clarity (e.g., who is “we”) - Response options: Add a “Not applicable” or “I don’t know” option; Be sure the scale is appropriate - General feedback: Survey may be better suited to large organizations with explicit PPE strategies/groups |
|  | Project | N = 6   - Timing of survey: Implemented too soon (unable to respond to some questions) - Survey questions: Some were unclear; Some were long and difficult to complete; Hard to respond on behalf of leadership of organization |
|  | Participant | N = 46   - General feedback: Good questions; Right length - Timing of survey: Too early after event to understand what impact will be - Relevance of survey: Not targeted to knowledge exchange events; May be better for one time events - Survey questions: Uncertainty re: trust question’s relevance; Importance of open ended questions, more may be needed; Be clear on what is being evaluated. What “activity” - Response options: Add a “not applicable” response option - Survey formatting: Number each question - Importance of follow-up: Questions only as good as what happens with the results |
