# Supplementary material for: Supporting the evaluation of public and patient engagement in health system organizations: Results from an implementation research study
Source: Health Expect. 2019 Aug 2;22(5):1132–43. doi: 10.1111/hex.12949 (PMC6803403; doi:10.1111/hex.12949)
Supplement: Supplementary file 2 [file HEX-22-1132-s002.docx]

**SUPPLEMENTARY MATERIAL: FEEDBACK SURVEY**

Thank you for completing this questionnaire. We would welcome your feedback on the survey itself. Please answer the following questions about the questionnaire you just completed.

1. Overall, the questionnaire was easy to use.

- Yes
- No

1. The instructions were clear and helpful.

- Yes
- No

1. The layout was easy to follow.

- Yes
- No

1. The questions were easy to understand.

- Yes
- No 🡪 please explain: ___________________________________________________

1. There were important questions missing.

- Yes 🡪 please explain: __________________________________________________
- No

1. Using this questionnaire will be useful for our organization.^1^

- Yes
- No 🡪 What would make it more useful? _____________________________________________________________________

1. Please identify one way in which this questionnaire can be improved:

|  |
| --- |

*^1^ Question 6 was not included in the participant survey.*
